# Fluid and Neuroimaging Biomarkers in Microgliopathy Colony‐Stimulating Factor‐1 Receptor‐Related Disorders

**DOI:** 10.1002/acn3.70250

**Published:** 2026-01-12

**Authors:** Tomasz Chmiela, Madison Reeves, Karen Jansen‐West, Judith Dunmore, Yuping Song, Audrey Strongosky, Sunil Gandhi, Gilana Pikover, Matt Blurton‐Jones, Robert C. Spitale, Erik H. Middlebrooks, Leonard Petrucelli, Mercedes Prudencio, Zbigniew K. Wszolek

**Affiliations:** ^1^ Department of Neurology Mayo Clinic Jacksonville Florida USA; ^2^ Department of Neurology, Faculty of Medical Sciences Medical University of Silesia Katowice Poland; ^3^ Department of Neuroscience Mayo Clinic Jacksonville Florida USA; ^4^ Savanna Biotherapeutics, Inc. Aliso Viejo California USA; ^5^ University of California Irvine California USA; ^6^ Department of Radiology Mayo Clinic Jacksonville Florida USA; ^7^ Neurobiology of Disease Graduate Program, Mayo Graduate School Mayo Clinic College of Medicine Jacksonville Florida USA

**Keywords:** biomarkers, CSF1R‐RD, microgliopathy

## Abstract

**Objective:**

This study aims to identify both fluid and neuroimaging biomarkers for CSF1R‐RD that can inform the optimal timing of treatment administration to maximize therapeutic benefit, while also providing sensitive quantitative measurements to monitor disease progression.

**Methods:**

Our study compared neuroimaging and fluid (plasma and cerebrospinal fluid (CSF)) biomarkers across three distinct populations: asymptomatic *CSF1R* pathogenic variant carriers (*N* = 14), symptomatic *CSF1R* pathogenic variant carriers (*N* = 17), and healthy controls (*N* = 30). We evaluated biomarker correlations with both an established (Montreal Cognitive Assessment (MoCA)) and a novel (CSF1R Clinical Severity Score (CCSS)) clinical diagnostic scale to investigate potential clinical utility. Additionally, we tested the relationship between select biomarkers and cortical thickness using 3D T1‐weighted MPRAGE scans, providing a highly valuable physiological component to our analyses.

**Results:**

Our results demonstrate that while plasma glial fibrillary acidic protein (GFAP) displays a high sensitivity for distinguishing early‐stage CSF1R‐RD patients from healthy controls, plasma neurofilament light chain (NfL) is more effective for tracking disease progression following the onset of symptoms.

**Interpretation:**

Overall, our study provides evidence for plasma NfL and GFAP as valuable biomarkers of earliest symptom onset and disease progression for CSF1R‐RD.

## 1 Introduction

Colony stimulating factor 1 receptor (CSF1R)‐related disorder (CSF1R‐RD) is a rare, hereditary, and rapidly progressive neurodegenerative disease caused by pathogenic variants in the *CSF1R* gene [1]. The *CSF1R* gene encodes a tyrosine kinase transmembrane receptor involved in the survival, proliferation, and differentiation of microglia [2, 3].

Pathological variants in the *CSF1R* gene cause primary microgliopathy that affects axon integrity leading to progressive and eventually fatal leukoencephalopathy. Pathologically, CSF1R‐RD is characterized by demyelination of cerebral white matter, frontoparietal lobe atrophy, thinning of the corpus callosum, swelling of axons, and pigmented glial cells [4].

Diagnostic criteria for CSF1R‐RD, established in 2017, aids in identifying patients with potential CSF1R‐RD; however, a definitive diagnosis requires the identification of a pathogenic variant in the CSF1R gene [5]. Only 25% of affected patients initially receive the correct diagnosis, with the most common misdiagnoses being frontotemporal dementia, multiple sclerosis, or non‐specific neurodegeneration/dementia [6]. Clinically, CSF1R‐RD manifests as personality and behavioral changes, dementia, and motor symptoms. The mean age of symptom onset is 40 ± 10 years in women and 47 ± 11 years in men [1]. In most of the affected individuals, rapid progression of disability is observed, leading quickly to total incapacitation [1, 7]. Besides the progression of motor symptoms, patients lose their ability to speak, perform voluntary movements, and eventually appear unaware of their surroundings. In the late stage, symptoms progress to a vegetative state [5, 6, 7]. No unified clinical scale currently exists that comprehensively captures the full spectrum of symptoms associated with the condition.

With a growing understanding of the pathophysiology of CSF1R‐RD [1, 8], therapeutic options are emerging, including the promising results of off‐label hematopoietic stem cell transplantation (HSCT) [9, 10]. Another promising option for asymptomatic *CSF1R* pathogenic variant carriers might be corticosteroid prophylaxis [8, 11]. This protective effect has been corroborated in a CSF1R‐RD animal model [12].

Neuroimaging plays a crucial role in the diagnosis of CSF1R‐RD and often prompts genetic testing in affected individuals. Typical radiologic findings in magnetic resonance imaging (MRI) include progressive bilateral white matter lesions, cerebral atrophy [13, 14]; and thinning of the corpus callosum. White matter calcifications have been observed in half of CSF1R‐RD [15] In 2012, Sundal et al. [16] developed a scale to assess the severity of changes in MRI dedicated to CSF1R‐RD.

Therefore, there is a growing interest in identifying precise and objective biomarkers that would allow for increased diagnostic sensitivity to help monitor patient status and to serve as a reference for evaluating treatment trials.

To address the current lack of robust biomarker data and the critical need for reliable diagnostic markers of disease progression, we leveraged a large cohort of *CSF1R* pathogenic variant carriers to conduct a comprehensive analysis of fluid, neuroimaging and clinical biomarkers. We assessed the potential of these biomarkers to reflect clinically detectable disease progression. We also developed a novel clinical diagnostic scale designed to capture the heterogeneous nature of CSF1R‐RD and to serve as a standardized and sensitive metric of clinical symptoms associated with CSF1R‐RD.

## 2 Methods

### 2.1 Study Design

Our observational study was comprised of 31 individuals, 11 males (35.5%) and 20 females (64.5%), with a mean age of 47.9 ± 15.9 years, ranging from 21 to 77 years of age, carrying pathogenic variants of *CSF1R*, including 17 symptomatic patients with a mean age of 49.4 ± 14.8 years and a mean age of symptom onset of 43.5 ± 16.0 years and 14 asymptomatic individuals with a mean age of 46.1 ± 17.5 years and 30 controls, 12 females (40%) and 18 males (60%), with a mean age of 51.4 ± 13.0 years. Cohort characteristics are summarized in Table 1. All asymptomatic and symptomatic patients were heterozygous *CSF1R* variant carriers. A list of *CSF1R* variants from this cohort is listed in Figure S1. Informed consent was obtained from all individuals according to standard protocols approved by the Mayo Clinic Institutional Review Board (IRB) ethics committee. The study protocol was approved by the Mayo Clinic IRB. Outliers were identified by examining individual datapoints that fell above or below the 1st or 99th quantile for each biomarker measurement and imputed with NA if the clinician deemed it likely the result of a technical error. Comprehensive details on neurological evaluations, neuroimaging procedures, and fluid biomarker assessments, along with a description of the statistical analyses, are provided in the Supporting Information.

**TABLE 1:** Descriptive statistics of the study cohort.

	CSF1R‐RD			Controls
	All	Asymptomatic	Symptomatic	
	*N* = 31	*N* = 14	*N* = 17	*N* = 30
Age at biomarker collection [mean ± SD (range)]	47.9 ± 15.9 (20.6–77.1)	46.1 ± 17.5 (20.6–77.1)	49.4 ± 14.8 (22.0–72.5)	51.4 ± 13.0 (31.7–75.0)
Age at symptom onset [mean ± SD (range)]	N/A	N/A	43.5 ± 16.0 (5, 7.1)	N/A
Sex [*n* (%)]				
Male	11 (35.5)	5 (35.6)	6 (35.3)	18 (60)
Female	20 (64.5)	9 (64.3)	11 (64.7)	12 (40)
CCSS total [median (IQR)]	5 (0, 29)	1 (0, 2.8)	29 (9.5, 47)	N/A
CCSS cognition [median (IQR)]	0 (0, 6)	0 (0, 0)	6 (0, 21.5)	N/A
CCSS affect and mood [median (IQR)]	0 (0, 1)	0 (0, 0)	1 (0, 5.5)	N/A
CCSS cranial nerves [median (IQR)]	0 (0, 0)	0 (0, 0)	0 (0, 0)	N/A
CCSS motor [median (IQR)]	4 (0, 14)	0 (0, 2)	14 (6.5, 25)	N/A
CCSS sensory [median (IQR)]	0 (0, 0)	0 (0, 0)	0 (0, 2)	N/A
CCSS other [median (IQR)]	0 (0, 2)	0 (0, 0)	2 (0, 3.5)	N/A
MoCA [median, (IQR)]	27 (15, 29)	28 (28, 30)	15 (11, 26.5)	N/A

Abbreviations: CCSS, CSF1R‐RD Clinical Severity Score; IQR, interquartile range; MoCA, Montreal Cognitive Assessment; *n*, number of cases; N/A, non applicable; SD, standard deviation.

## 3 Results

### 3.1 Study Cohort

We leveraged a robust cohort of 17 symptomatic and 14 asymptomatic *CSF1R* pathogenic variant carriers and 30 healthy controls (*N* = 61) to identify candidate biomarkers capable of guiding the optimal timing of therapeutic intervention for presymptomatic carriers transitioning to symptomatic states, and enabling the sensitive monitoring of disease progression after symptom onset. The 31 *CSF1R* pathogenic carriers (11 males, 35.5%; 20 females, 64.5%) had a mean age of 47.9 ± 15.9 years (range 21–77 years). Individuals were identified as asymptomatic (mean age: 46.1 ± 17.5 years, range 21–77 years) based on the presence of a genetically confirmed *CSF1R* pathogenic variant and no detectable clinical symptoms associated with CSF1R‐RD. Individuals were classified as symptomatic (mean age: 49.4 ± 14.8 years) given the presence of a pathogenic variant along with a clinically detectable symptom onset (mean age of symptom onset: 43.5 ± 16.0 years; no statistically significant differences in age at symptom onset were observed between sexes), ranging from the presence of cognitive symptoms, mood impairment, behavioral changes, motor symptoms like parkinsonism, cerebellar and pyramidal signs, and sensory dysfunction. In the studied cohort, 11 out of 17 individuals (64.7%) exhibited cognitive symptoms, ranging from mild cognitive impairment to dementia. Behavioral changes were observed in 7 individuals (41.2%). Motor symptoms were present in 14 individuals (82.4%), including parkinsonism (70.5%), increased muscle tone (76.5%)—typically a combination of spasticity and rigidity—along with pyramidal (58.8%) and cerebellar signs (47.1%) and gait abnormalities (76.5%).

The control group consisted of 30 healthy individuals negative for *CSF1R* pathogenic variants (18 males, 60%; 12 females, 40%) with a mean age of 51.4 ± 13.0 years (range 32–75 years). Twenty‐three participants with *CSF1R* pathogenic variant carriers (13 asymptomatic, and 10 symptomatic) had 3‐Tesla MRI of the brain available, obtained within one week of biomarker collection.

### 3.2 CSF1R‐RD Patients Show Significant White Matter Atrophy

**FIGURE 1:**
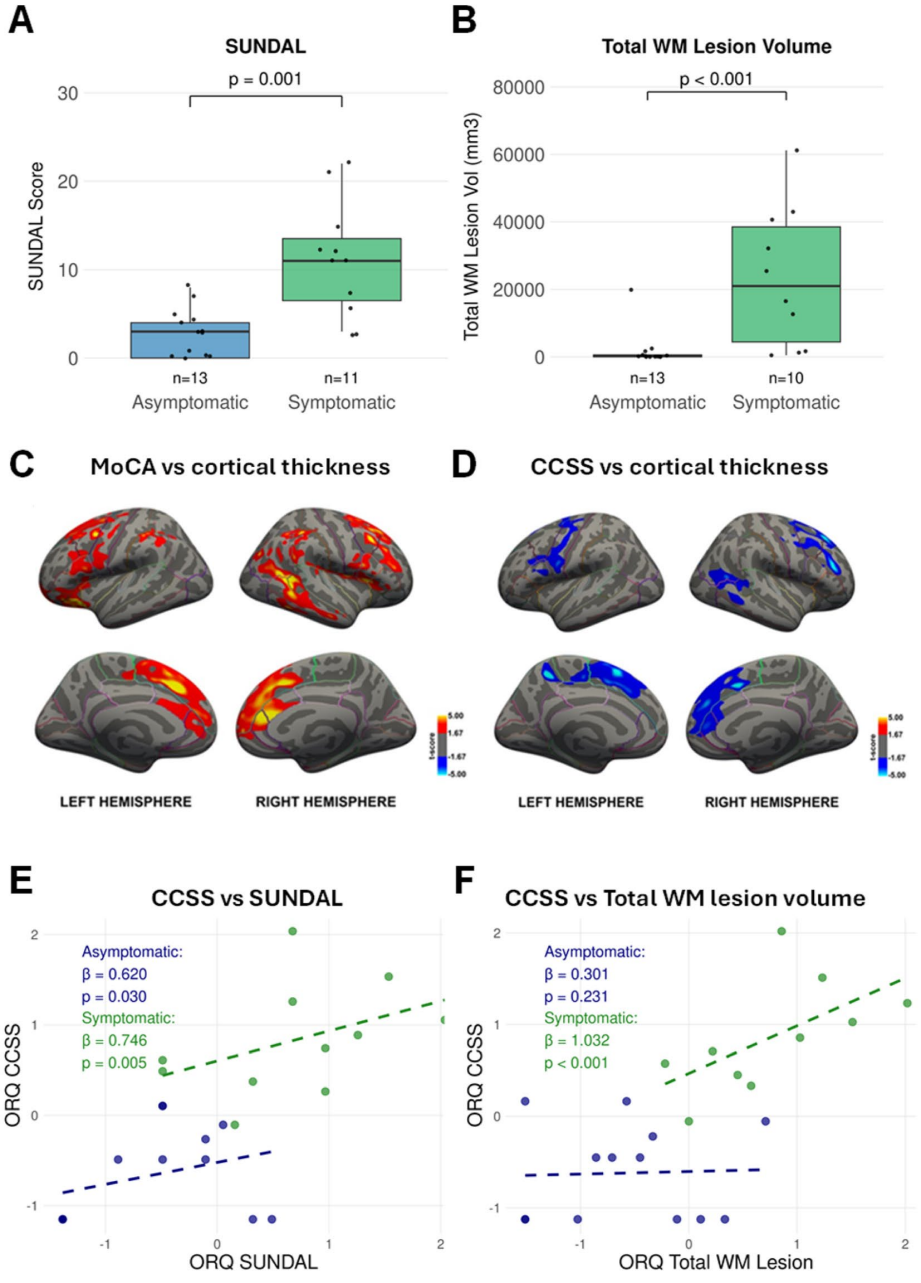
Symptomatic patients reflect higher clinical diagnostic severity and physiological changes. Sundal scores (A) and total white matter lesion volume (B) are significantly elevated in symptomatic patients compared to asymptomatic patients. Correlations of MoCA (C) and CCSS (D) with cortical thickness measured with 3D T1‐weighted MPRAGE scans across multiple brain regions indicate that both clinical assessments are reflective of the physiological changes associated with CSF1R‐RD disease progression. (E, F) Scatterplots of ranked relationships (ORQ‐transformed) between CSF1R‐RD Clinical Severity Score (CCSS) and Sundal scores (E) or total white matter lesion volume (F).

Clinical diagnostic severity was calculated from neuroimaging MRI scans using the Sundal scale [16] (Table 2). Symptomatic patients were characterized by significantly higher total Sundal scale scores [11 (6.5, 13.5) points] compared to asymptomatic [3 (0, 4) points] individuals (Figure 1A, Table 2). Analysis of individual Sundal subscoring components showed that the atrophy component was elevated in symptomatic patients [4 (0.5, 4) points] compared to asymptomatic individuals [0 (0, 0) points] (Figure S2A and Table 2). Notably, the white matter subscoring component was even more significantly elevated in symptomatic patients [7 (6.5, 9.5) points] when compared with asymptomatic individuals [3 (0, 4) points] (Figure S2B and Table 2). Symptomatic patients also exhibited substantially higher white matter lesion volume [20,995 (4418, 38,488) mm^3^] compared to asymptomatic patients [179 (12, 573) mm^3^] (Figure 1B and Table 2). Multiple neuroimaging markers showed trends toward reductions in symptomatic patients compared to asymptomatic patients, including normalized brain volume [0.73 (0.69, 0.79) vs. 0.79 (0.78, 0.81) mm^3^; *p* = 0.034] (Table 2 and Figure S2C), normalized white matter volume [0.27 (0.26, 0.32) vs. 0.32 (0.30, 0.32) mm^3^; *p* = 0.077] (Table 2 and Figure S2D), corpus callosum volume [2568.6 (1693.2, 3471.2) vs. 3528.5 (3372.3, 3810.8) mm^3^; *p* = 0.057] (Table 2 and Figure S2E), cerebral white matter volume [421.6 (388.5, 453.2) vs. 481.3 (433.9, 503.4) cm^3^; *p* = 0.166] (Table 2 and Figure S2F), subcortical gray matter volume [52.8 (49.8, 56.2) vs. 60.0 (57.6, 61.3) cm^3^; *p* = 0.067] (Table 2 and Figure S2G), and toward an increase in ventricular volume [56.1 (39.9, 68.8) vs. 20.8 (18.2, 27.3) cm^3^; *p* = 0.015] (Table 2 and Figure S2H). However, none of these differences reached the Bonferroni‐corrected significance threshold (*p* < 0.0045). Cortex volume did not show any substantial difference between symptomatic and asymptomatic patients [458.0 (446.6, 521.2) vs. 500.1 (462.9, 532.7) cm^3^; *p* = 0.483] (Table 2 and Figure S2I).

**TABLE 2:** Differences in white matter lesions and atrophy, as well as biofluid biomarkers, between healthy controls, asymptomatic and symptomatic *CSF1R* mutation carriers.

	Asymptomatic [median (IQR)]	Symptomatic [median (IQR)]	Healthy controls [median (IQR)]	*p*
*MRI*				
Sundal scale				
Total	3 (0–4)	11 (6.5–13.5)	N/A	0.001
White matter (WM)	3 (0–4)	7 (6.5–9.5)	N/A	< 0.001
Atrophy	0 (0–0)	4 (0.5–4)	N/A	0.007
Total WM lesion volume (mm^3^)	179 (12, 573)	20,995 (4418, 38,488)	N/A	< 0.001
Normalized brain volume	0.79 (0.78, 0.81)	0.73 (0.69, 0.79)	N/A	0.034
Normalized WM volume	0.32 (0.30, 0.32)	0.27 (0.26, 0.32)	N/A	0.077
Corpus callosum volume (mm^3^)	3528.5 (3372.3, 3810.8)	2568.6 (1693.2, 3471.2)	N/A	0.057
Cortex volume (cm^3^)	500.1 (462.9, 532.7)	458.0 (446.6, 521.2)	N/A	0.483
Cerebral WM volume (mm^3^)	481.3 (433.9, 503.4)	421.6 (388.5, 453.2)	N/A	0.166
Subcortical gray matter volume (cm^3^)	60.0 (57.6, 61.3)	52.8 (49.8, 56.2)	N/A	0.067
Ventricles volume (cm^3^)	20.8 (18.2, 27.3)	56.1 (39.9, 68.8)	N/A	0.015
*Plasma biomarkers*				
M‐CSF (pg/mL)	29.8 (26.4, 36.3)	31.4 (26.4, 35.2)	23.8 (21.0, 27.4)	0.002
IL‐34 (pg/mL)	2.7 (1.7, 3.1)	2.5 (2.0, 3.2)	1.5 (1.1, 1.9)	< 0.001
NfL (pg/mL)	6.2 (4.5, 13.1)	38.5 (14.8, 102.9)	6.6 (4.4, 9.6)	< 0.001
Osteopontin (pg/mL)	3871.3 (2861.1, 4848.7)	3327.0 (2481.2, 4351.5)	4087.8 (3100.2, 4712.7)	0.540
GFAP (pg/mL)	181.1 (128.9, 246.5)	280.0 (177.8, 689.4)	89.3 (70.5, 154.2)	< 0.001
*CSF biomarkers*				
M‐CSF, (pg/mL)	7.9 (6.8, 10.4)	13.3 (10.8, 15.8)	5.5 (4.2, 6.7)	< 0.001
IL 34, (pg/mL)	142.5 (123.6, 151.6)	132.3 (95.2, 224.4)	58.2 (46.1, 71.7)	< 0.001
NfL, (pg/mL)	505.0 (287.6, 612.9)	2972.4 (2205.2, 4646.9)	489.2 (381.6, 612.8)	0.019
Osteopontin (pg/mL)	12,723.8 (9835.2, 17,367.7)	16,578.9 (7117.5, 24,452.2)	16,949.1 (8400.4, 21,772.9)	0.797
GFAP (pg/mL)	12,102.5 (9129.2, 14,654.7)	20,920.3 (10,805.1, 24,752.7)	8405.1 (5628.6, 11,631.5)	0.007

*Note:* Mann–Whitney *U* tests were employed for MRI level comparisons due to violation of normality assumptions. For Mann–Whitney *U* tests, *p*‐values < 0.0045 were considered significant after applying a Bonferroni correction for multiple testing. Kruskal‐Wallis tests were employed for plasma and CSF level comparisons due to violation of normality assumptions. For Kruskal‐Wallis tests, *p*‐values < 0.01 were considered significant after applying a Bonferroni correction for multiple testing.

Abbreviations: CSF, cerebrospinal fluid; GFAP, glial fibrillary acidic protein; IL‐34, interleukin‐34; IQR, interquartile range; M‐CSF, macrophage colony stimulating factor; N/A, not applicable; NfL, neurofilament light chain; WM, white matter.

### 3.3 CSF1R‐RD Patients Show Changes in MoCA and CCSS

The Montreal Cognitive Assessment (MoCA) is a well‐established clinical diagnostic tool used to track the severity of cognitive decline in patients. Patients begin with a score of 30 and lose points as they lose cognitive function. Cognition of symptomatic CSF1R‐RD patients was affected in our study cohort with a median score of 15, while asymptomatic individuals were virtually unaffected with a median score of 28 (Table 1). To affirm the informative ability of MoCA in CSF1R‐RD patients, we obtained cortical thickness measurements using 3D T1‐weighted MPRAGE imaging. Higher MoCA scores associated with higher cortical thickness across multiple brain regions, including the bilateral prefrontal regions, cingulum, and inferior parietal lobe, as well as the right lateral temporal lobe (Figure 1C). Moreover, higher MoCA scores correlated with lower total white matter lesion volume (*r* = −0.60, *p* = 0.003), lower Sundal scores (*r* = −0.51, *p* = 0.012), and higher corpus callosum volume (*r* = 0.53, *p* = 0.011) (Table 3). In contrast, normalized brain volumes (*r* = 0.44, *p* = 0.040), subcortical gray matter volume (*r* = 0.34, *p* = 0.118), normalized white matter volume (*r* = 0.37, *p* = 0.087), cortex volume (*r* = 0.19, *p* = 0.393), cerebral white matter volume (*r* = 0.23, *p* = 0.306), ventricle volume (*r* = −0.46, *p* = 0.032) did not correlate with MoCA scoring (Table 3).

**TABLE 3:** Correlation between brain lesion volume, brain atrophy, fluid biomarker levels and clinical severity diagnostics.

	MoCA	CCSS
	Spearman's *r*, *p*	Spearman's *r*, *p*
MRI		
Sundal score	−0.51, 0.012	0.69, < 0.001
Total WM Lesion Volume (mm^3^)	−0.60, 0.003	0.70, < 0.001
Normalized Brain Volume	0.44, 0.040	−0.65, < 0.001
Normalized WM Volume	0.37, 0.087	−0.46, 0.028
Corpus Callosum Total Volume (mm^3^)	0.53, 0.011	−0.64, 0.001
Cortex Volume (mm^3^)	0.19, 0.393	−0.47, 0.024
Cerebral WM Volume (mm^3^)	0.23, 0.306	−0.46, 0.029
Subcortical Gray Matter Volume (mm^3^)	0.34, 0.118	−0.65, < 0.001
Ventricles Volume (mm^3^)	−0.46, 0.032	0.48, 0.020
Plasma biomarkers		
M‐CSF (pg/mL)	−0.14, 0.486	0.16, 0.432
IL34 (pg/mL)	−0.14, 0.498	0.27, 0.172
NfL (pg/mL)	−0.61, < 0.001	0.76, < 0.001
Osteopontin (pg/mL)	−0.06, 0.772	0.20, 0.308
GFAP (pg/mL)	−0.49, 0.007	0.67, < 0.001
CSF biomarkers		
M‐CSF (pg/mL)	−0.38, 0.158	0.53, 0.043
IL34 (pg/mL)	0.11, 0.723	−0.22, 0.484
NfL (pg/mL)	−0.64, 0.010	0.74, 0.002
Osteopontin (pg/mL)	−0.34, 0.275	0.37, 0.236
GFAP (pg/mL)	−0.49, 0.063	0.63, 0.011

*Note:* Spearman's test of correlation selected for untransformed, non‐normal data. After applying a Bonferroni correction for multiple testing, *p* values < 0.025 were considered significant.

Abbreviations: CCSS, CSF1R‐RD Clinical Severity Score; GFAP, glial fibrillary acidic protein; IL‐34, interleukin‐34; M‐CSF, macrophage colony stimulating factor; NfL, neurofilaments light chains; WM, white matter.

Next, to better evaluate symptom severity in CSF1R‐RD patients, we developed the CSF1R Clinical Severity Score (CCSS), designed to maximize sensitivity to symptom progression. The CCSS is a semi‐quantitative scale that assesses the patient in five domains: cognition, mood and affect, cranial nerves, motor function and sensory function, and is sensitive enough to detect even minor cognitive and motor deviations. Scores range from 0 (unaffected) to 244 (severe) (Tool S1). Symptomatic *CSF1R* carriers showed median CCSS total values of 29, scoring most strongly with worsened cognition and motor function (Table 1). To determine the utility of the CCSS, we also compared it to the neuroanatomical changes observed using 3D T1‐weighted MPRAGE scans. CCSS showed an inverse correlation with cortical thickness, with poor CCSS scores associated with cortical thinning across broad frontal areas, the right precuneus, left inferior parietal, and middle temporal regions (Figure 1D). CCSS also positively correlated with Sundal score (*r* = 0.69, *p* < 0.001, Figure 1E), total white matter lesion volume (*r* = 0.70, *p* < 0.001, Figure 1F), and lower corpus callosum volume (*r* = −0.64, *p* = 0.001) (Table 3). Furthermore, CCSS was significantly associated with lower subcortical gray matter (*r* = −0.65, *p* < 0.001) and cortex (*r* = −0.47, *p* = 0.024) volumes, and increased ventricle volumes (*r* = 0.48, *p* = 0.020) (Table 3). These findings support the functionality of our novel CCSS tool for monitoring disease progression in CSF1R‐RD patients.

### 3.4 Elevated Fluid Biomarkers in CSF1R‐RD Patients

**FIGURE 2:**
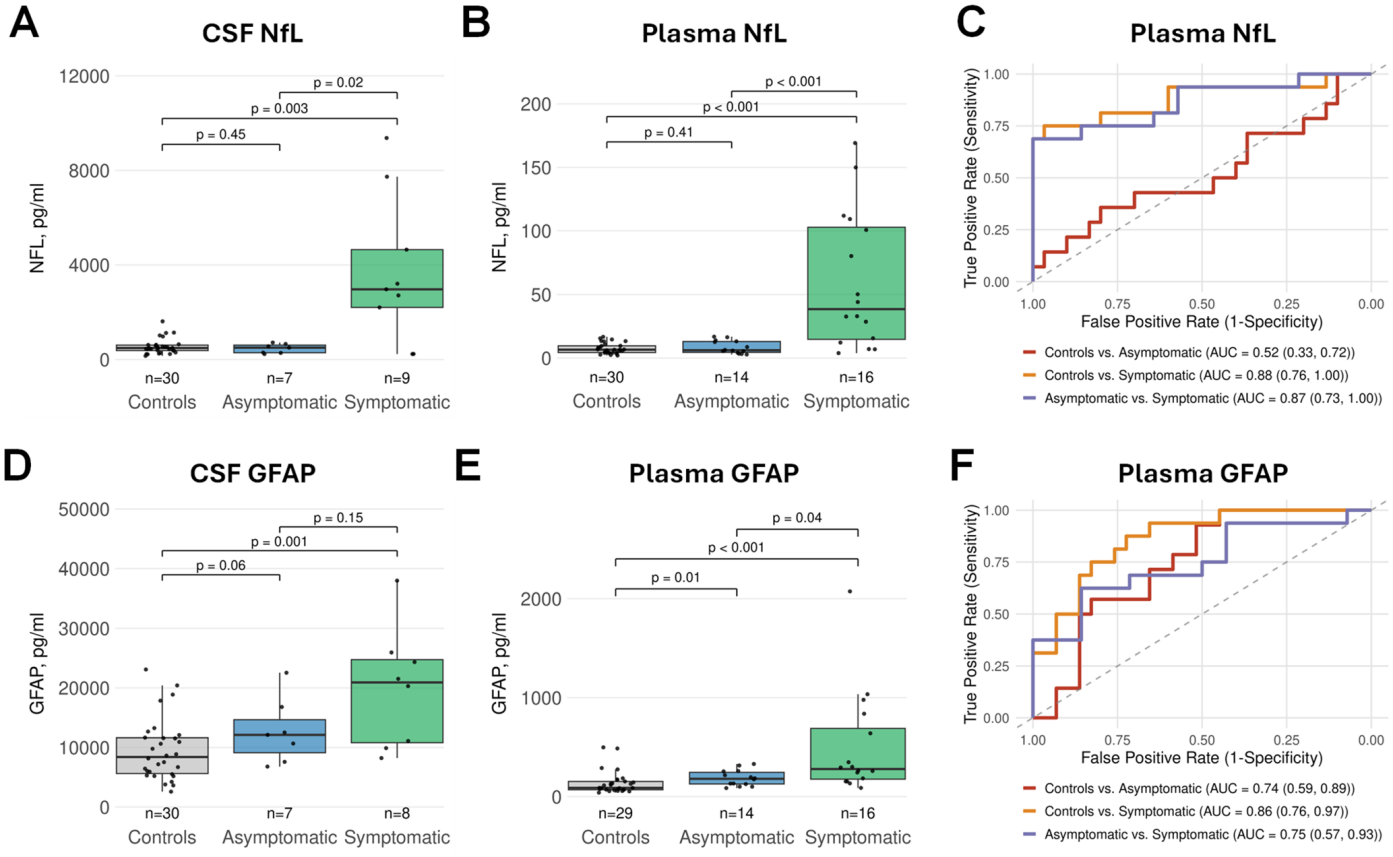
NfL and GFAP levels and discriminatory ability between symptomatic CSF1R‐RD patients, asymptomatic *CSF1R* carriers, and controls. (A, B) NfL levels in CSF (A) and plasma (B), compared between healthy controls, asymptomatic, and symptomatic pathogenic *CSF1R* mutation carriers using Dunn's tests (with Bonferroni‐corrected significance threshold *p* < 0.0167). (C) Receiver operating curves (ROC) with area under the curve (AUC) measure (with 95% confidence interval) demonstrate the ability of plasma NfL to discriminate between symptomatic, asymptomatic, and control groups. (D, E) GFAP levels in CSF (D) and plasma (E), compared between healthy controls, asymptomatic, and symptomatic pathogenic *CSF1R* mutation carriers using Dunn's tests (with Bonferroni‐corrected significance threshold *p* < 0.0167). (F) Receiver operating curves (ROC) with area under the curve (AUC) measure (with 95% confidence interval) test the ability of plasma GFAP to discriminate between symptomatic, asymptomatic, and control groups.

Given the critical need for identifying biomarkers that may aid in assessing severity and progression for CSF1R‐RD patients, we interrogated established biomarkers of neuronal injury (NfL) [17, 18, 19], blood–brain barrier breaching through astrocytic damage (GFAP) [20, 21, 22], CSF1R ligand receptor proteins (M‐CSF, IL‐34) [23, 24, 25], and inflammation (osteopontin) [26], some of which may have been evaluated in smaller cohorts of CSF1R‐RD patients (Table S1). We measured these markers in both cerebrospinal fluid (CSF) and plasma in all subjects. In CSF, NfL levels were elevated in symptomatic *CSF1R* carriers [2972.4 (2205.2, 4646.9) pg/mL] compared to controls [489.2 (381.6, 612.75) pg/mL] (Figure 2A). CSF NfL levels in symptomatic *CSF1R* carriers were also elevated compared to asymptomatic carriers [505.0 (287.6, 612.9) pg/mL], but significance bordered the Bonferroni‐corrected significance threshold (*p* < 0.01). In plasma, NfL levels were significantly elevated in symptomatic *CSF1R* carriers [38.5 (14.8, 102.9) pg/mL] compared to both asymptomatic *CSF1R* carriers [6.2 (4.5, 13.1) pg/mL] and healthy controls [6.6 (4.4, 9.6) pg/mL] (Figure 2B; *p* < 0.001). To assess the ability of NfL levels to distinguish between our study groups, we created receiver operator characteristic (ROC) curves. In doing so, plasma NfL [AUC = 0.88 (0.76, 1.00)] (Figure 2C), and to a lesser extent CSF NfL [AUC = 0.80 (0.55, 1.00)] (Figure S3A), strongly differentiated between symptomatic cases and controls. In contrast, neither CSF [AUC = 0.52 (0.25, 0.78)] (Figure S3A) nor plasma NfL [AUC = 0.52 (0.33, 0.72)] (Figure 2C) levels differentiated asymptomatic from controls.

Interestingly, CSF GFAP was increased in a stepwise manner from controls [8405.1 (5628.6, 11,631.5) pg/mL], to asymptomatic [12,102.5 (9129.2, 14654.7) pg/mL], and symptomatic [20,920.3 (10,805.1, 24,752.7) pg/mL] *CSF1R* carriers (Figure 2D). Notably, the significance of increased GFAP levels was stronger in plasma, showing a clearer stepwise increase from controls [89.3 (70.5, 154.2) pg/mL], to asymptomatic [181.1 (128.9, 246.5) pg/mL], and symptomatic [280.0 (177.8, 689.4) pg/mL] *CSF1R* carriers (Figure 2D). Further, plasma GFAP [AUC = 0.86 (0.76, 0.97)] (Figure 2F), and to a lesser extent CSF GFAP [AUC = 0.83 (0.67, 0.99)] (Figure S3B), significantly distinguished symptomatic *CSF1R* carriers from healthy controls. Importantly, while plasma NfL levels were not able to successfully differentiate asymptomatic *CSF1R* carriers from controls (Figure 2C), plasma [AUC = 0.74 (0.59, 0.89)] (Figure 2F), and to a lesser extent CSF [AUC = 0.70 (0.51, 0.90)] (Figure S3B), GFAP levels served to more reliably distinguish between asymptomatic cases and controls.

We also measured M‐CSF, IL‐34, and osteopontin in CSF and plasma. M‐CSF levels were elevated in symptomatic patients [CSF: 13.3 (10.8, 15.8), plasma: 31.4 (26.4, 35.2), pg/mL] compared to controls [CSF: 5.5 (4.2, 6.7), plasma: 23.8 (21.0, 27.4), pg/mL] (Figure S4A,B), as well as when comparing asymptomatic individuals [CSF: 7.9 (6.8, 10.4), plasma: 29.8 (26.4, 36.3) pg/mL] to controls [CSF: 5.5 (4.2, 6.7), plasma: 23.8 (21.0, 27.4) pg/mL] (Figure S4A,B). Similar findings were observed for IL‐34, where significantly higher levels were observed in symptomatic [CSF: 132.3 (95.2, 224.4), plasma: 2.5 (2.0, 3.2), pg/mL] and asymptomatic [CSF: 142.5 (123.6, 151.6), plasma: 2.7 (1.7, 3.1), pg/mL] individuals compared to controls [CSF: 58.2 (46.1, 71.7), plasma: 1.5 (1.1, 1.9), pg/mL] (Figure S4C,D). However, there were no differences in either M‐CSF or IL‐34 levels between symptomatic and asymptomatic individuals (Figure S4A–D). Furthermore, osteopontin levels did not differ significantly between symptomatic, asymptomatic, and healthy control groups in either CSF or plasma (Figure S4E,F). Overall, our data suggest that plasma GFAP may be more sensitive to early pathological changes preceding symptom onset, while plasma NfL better reflects disease progression after initial symptom onset.

### 3.5 GFAP and NfL Biomarkers May Inform on Clinical and Neuroanatomical Changes in CSF1R‐RD Patients

**FIGURE 3:**
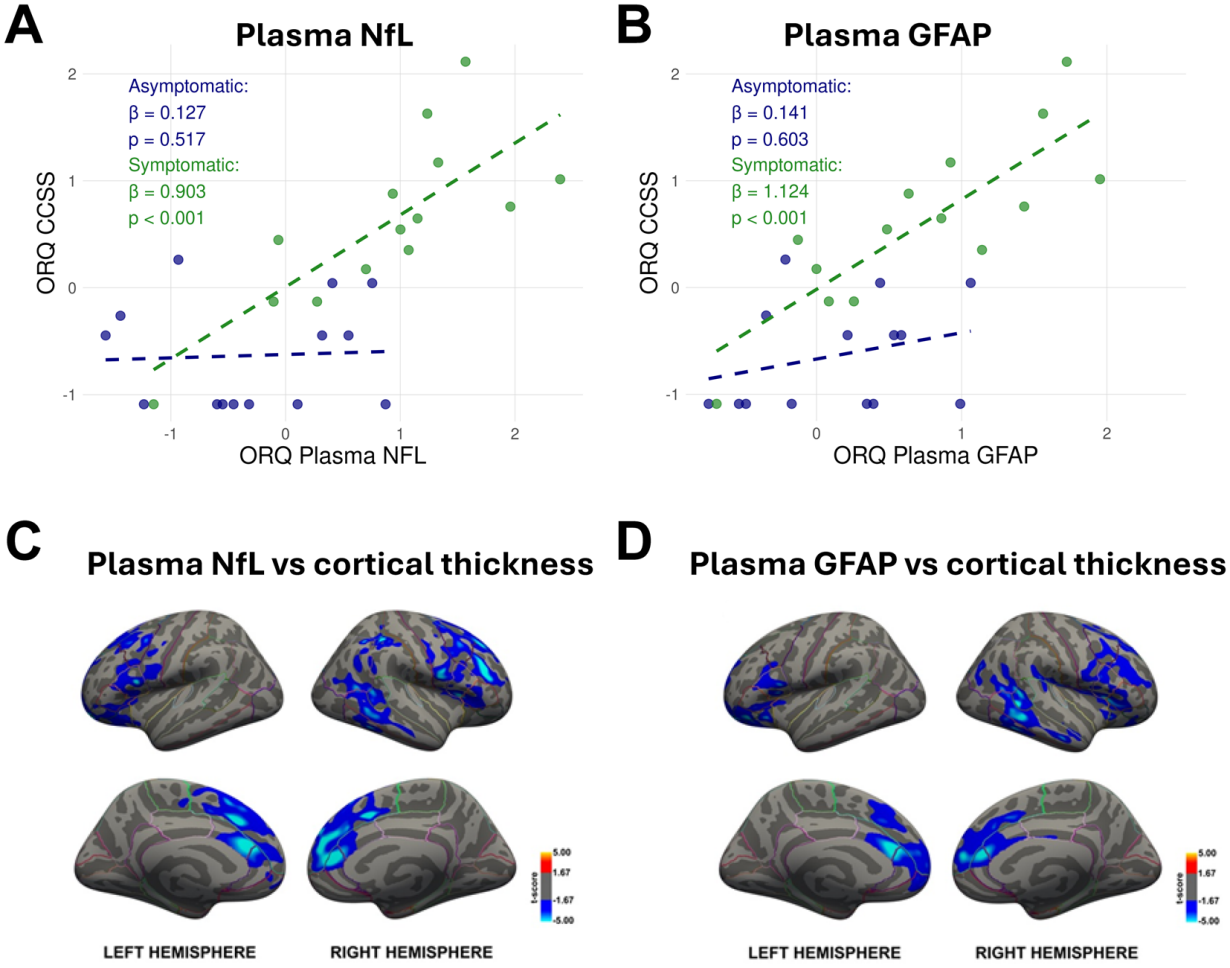
Plasma NfL and GFAP sensitively reflect CCSS and physiological changes associated with CSF1R‐RD. (A, B) Scatterplots of ranked relationships (ORQ‐transformed) between CSF1R‐RD Clinical Severity Score (CCSS) and plasma NfL (A) and GFAP (B) indicate a strong positive relationship between these biomarkers and CCSS in symptomatic patients. (C, D) Correlations of plasma NfL (C) and plasma GFAP (D) with cortical thickness measured with 3D T1‐weighted MPRAGE scans across multiple brain regions indicate that both biomarkers are reflective of the physiological changes associated with CSF1R‐RD disease progression.

To determine if fluid biomarkers inform clinical assessments, we performed associations between NfL and GFAP with MoCA and CCSS. NfL demonstrated the most robust correlations with CCSS in both plasma (*r* = 0.76, *p* < 0.001) (Figure 3A) and CSF (*r* = 0.74, *p* = 0.002) (Table 3), but also with MoCA to a lesser degree [plasma: (*r* = −0.61, *p* < 0.001), CSF: (*r* = −0.64, *p* = 0.010)] (Table 3). Increased GFAP levels were also highly associated with worsened CCSS especially in plasma (*r* = 0.67, *p* < 0.001) (Figure 3B), and in CSF (*r* = 0.63, *p* = 0.011) (Table 3). Plasma GFAP (*r* = −0.49, *p* = 0.007), but not CSF GFAP (*r* = −0.49, *p* = 0.063), also associated with MoCA scores (Table 3). Weaker to no correlations were observed between other fluid biomarkers (M‐CSF, IL‐34, osteopontin) and either MoCA or CCSS scores (Table 3).

Next, we evaluated the relationships between the levels of fluid biomarkers and cortical thickness measurements from 3D T1‐weighted MPRAGE scans. Interestingly, plasma NfL was inversely correlated with cortical thickness across broad prefrontal regions, cingulum, precentral gyrus, as well as the right inferior parietal lobe and lateral temporal lobe (Figure 3C). Additionally, plasma NfL correlated strongly with Sundal score (*r* = 0.71, *p* < 0.001), and showed higher total white matter lesion volume (*r* = 0.56, *p* = 0.005), and lower normalized brain volumes (*r* = −0.70, *p* < 0.001) (Table 4). CSF NfL showed even stronger correlations with Sundal score (*r* = 0.85, *p* < 0.001) and total white matter lesion volume (*r* = 0.79, p < 0.001), while showing a trend toward correlation with normalized brain volume (*r* = −0.66, *p* = 0.012), but not meeting the Bonferroni‐corrected significance threshold of *p* < 0.01 (Table 4).

**TABLE 4:** Testing the correlation between fluid biomarkers and neuroanatomical changes.

	Plasma					CSF				
	M‐CSF	IL 34	NfL	Osteopontin	GFAP	M‐CSF	IL 34	NfL	Osteopontin	GFAP
	Spearman's *r*, *p*					Spearman's *r*, *p*				
Sundal score	−0.22, 0.308	0.18, 0.405	0.71, < 0.001	0.04, 0.859	0.36, 0.088	0.40, 0.153	−0.61, 0.04	0.85, < 0.001	0.59, 0.058	0.71, 0.004
Sundal score (WM)	−0.22, 0.308	0.24, 0.250	0.69, < 0.001	0.09, 0.670	0.36, 0.087	0.43, 0.125	−0.64, 0.035	0.86 < 0.001	0.71, 0.014	0.74, 0.003
Sundal score (atrophy)	−0.08, 0.694	0.06, 0.790	0.72, < 0.001	−0.06, 0.781	0.40, 0.056	0.51, 0.064	−0.29, 0.380	0.71, 0.004	0.39, 0.241	0.64, 0.013
Total WM lesion volume (mm3)	−0.15 0.496	−0.02 0.925	0.56 0.005	−0.15 0.512	0.31, 0.156	0.30, 0.293	−0.64, 0.033	0.79, < 0.001	0.69, 0.023	0.65, 0.012
Normalized brain volume	0.14, 0.530	0.02, 0.938	−0.70, < 0.001	0.03, 0.889	−0.44, 0.038	−0.49, 0.081	0.51, 0.114	−0.66, 0.012	−0.36, 0.273	−0.53, 0.057
Normalized WM volume	0.10, 0.655	−0.10, 0.642	−0.57, 0.005	0.29, 0.188	−0.32, 0.134	−0.38, 0.177	−0.06, 0.863	−0.37, 0.193	−0.32, 0.339	−0.40, 0.156
Corpus callosum total volume (mm^3^)	−0.06, 0.771	−0.31, 0.154	−0.55, 0.007	0.10, 0.650	−0.33, 0.130	−0.33, 0.253	0.15, 0.673	−0.40, 0.160	−0.20, 0.558	−0.35, 0.221
Cortex volume (mm^3^)	−0.32, 0.142	−0.11, 0.630	−0.56, 0.006	−0.24, 0.288	−0.32, 0.136	−0.38, 0.186	−0.07, 0.839	−0.27, 0.349	−0.26, 0.435	−0.20, 0.483
Cerebral WM volume (mm^3^)	−0.32, 0.133	−0.24, 0.261	−0.61, 0.002	−0.11, 0.639	−0.27, 0.206	−0.50, 0.072	−0.26, 0.435	−0.16, 0.584	−0.19, 0.576	−0.31, 0.288
Subcortical gray matter volume (mm^3^)	−0.23, 0.293	−0.18, 0.400	−0.80, < 0.001	−0.16, 0.466	−0.49, 0.019	−0.58, 0.032	−0.05, 0.882	−0.46, 0.101	−0.37, 0.261	−0.53, 0.057
Ventricles volume (mm^3^)	−0.23, 0.287	−0.29, 0.178	0.62, 0.002	−0.12, 0.593	0.43, 0.041	0.50, 0.069	−0.59, 0.061	0.80, < 0.001	0.31, 0.356	0.67, 0.011

*Note:* Spearman's test of correlation selected for untransformed, non‐normal data. After applying a Bonferroni correction for multiple testing, *p*‐values < 0.01 were considered significant.

Abbreviations: CSF, cerebrospinal fluid; GFAP, glial fibrillary acidic protein; IL‐34, interleukin‐34; M‐CSF, macrophage colony stimulating factor; NfL, neurofilaments light chains; WM, white matter.

Plasma GFAP levels were also inversely correlated with cortical thickness in the bilateral frontal operculum, cingulum, superior frontal gyrus, as well as the right inferior parietal and lateral temporal lobes (Figure 3D). Plasma GFAP showed a trend toward correlation with Sundal score (*r* = 0.36, *p* = 0.088) and smaller normalized brain volumes (*r* = −0.44, *p* = 0.038), but did not reach significance with total white matter lesion volume (*r* = 0.31, *p* = 0.156) (Table 4). CSF GFAP also demonstrated strong correlations with Sundal score (*r* = 0.71, *p* = 0.004), but showed only a trend toward correlation with total white matter lesion volume (*r* = 0.65, *p* = 0.012) and normalized brain volume (*r* = −0.53, *p* = 0.057) (Table 4). Of note, the levels of plasma or CSF for M‐CSF, IL‐34, and osteopontin exhibited weak to no associations with Sundal scores or white matter lesions (Table 4). Overall, plasma NfL and GFAP showed the strongest associations with CCSS and MoCA clinical assessments, as well as with neuroanatomical changes including cortical thinning, normalized brain volumes, Sundal scoring, and total white matter lesions. Taken together, these findings provide strong evidence to support the utility of plasma NfL and GFAP as biomarkers for tracking CSF1R‐RD disease progression, with plasma GFAP displaying the additional capability of identifying the earliest stages of disease.

## 4 Discussion

Our study leveraged a well‐characterized cohort of 31 individuals carrying a pathogenic *CSF1R* variant, representing, to our knowledge, the largest cohort of its kind to date. All individuals underwent fluid biomarker assessment, with 23 undergoing additional neuroimaging biomarker testing. Additionally, we developed a novel clinical diagnostic tool, the CSF1R‐RD Clinical Severity Score (CCSS), which we believe is an accurate, reproducible, and sensitive scale for monitoring symptoms in patients with CSF1R‐RD. This study aimed to identify both fluid and neuroimaging biomarkers for CSF1R‐RD that can inform the optimal timing of treatment administration to maximize therapeutic benefit, while also providing sensitive quantitative measurements to monitor disease progression.

Neuroimaging biomarkers have been extensively studied in CSF1R‐RD research, with some accurately reflecting established pathological features of the disease. However, none have demonstrated sufficient reliability in identifying the critical transition from presymptomatic to symptomatic stages. Further, the sensitivity of neuroimaging biomarkers to capturing nuanced changes in disease progression remains uncertain and continues to be a subject of debate. Another important limitation to the utility of neuroimaging biomarkers in CSF1R‐RD is the significant overlap in imaging features with other leukoencephalopathies [13, 16]. In our study, symptomatic patients had significantly higher Sundal scores, and were characterized by more severe white matter lesions and brain atrophy measured by Sundal subscales. White matter lesions were highly elevated in symptomatic patients compared with asymptomatic patients, suggesting that white matter involvement in CSF1R‐RD pathology may be a marker for conversion to the symptomatic phase. In our cohort, we also found a significant correlation between the severity of neuroimaging changes and the clinical symptoms of the disease, as assessed by the CCSS and MoCA. Notably, we found that reduced cortical thickness, most prominently across the prefrontal cortex, is significantly correlated with poor clinical status. This result supports work by Kinoshito et al. [27], which demonstrated that MR images were associated with more advanced pathological lesion stages. As demonstrated in this paper, the Sundal scale [16] has strong potential for describing the clinical status in CSF1R‐RD; however, its semi‐quantitative nature may limit its utility for detecting subtle changes in longitudinal cohorts, in which case radiologic assessment based on volume measurements is likely to be superior. When assessing asymptomatic patients, it is also important to consider that white matter changes may occur during the preclinical period of CSF1R‐RD or may be attributed to other causes.

Fluid biomarkers offer significant advantages over neuroimaging biomarkers for tracking CSF1R‐RD progression and detecting early symptom onset. They offer a high degree of reliability, reproducibility, and can be collected through minimally invasive procedures, making them far more practical for routine clinical use. Additionally, fluid biomarkers enable more frequent and accessible testing, which is critical for capturing more subtle changes in disease progression and creating an optimized care plan for CSF1R‐RD patients. So far, basic CSF analyses have proven to be unremarkable in the monitoring of CSF1R‐RD [28]. Consequently, there remains an urgent need to identify alternative fluid biomarkers that can accurately reflect CSF1R‐RD disease progression.

NfL is well‐established as a robust biomarker for other rapidly progressive neurodegenerative diseases, but it is known to lack disease specificity [17, 19, 29]. Previous work by Hayer et al. explored the utility of NfL as a robust fluid biomarker for CSF1R‐RD and found elevated levels of NfL in both CSF and plasma in symptomatic patients [30]. Notably, they showed that NfL can differentiate CSF1R‐RD from multiple sclerosis (MS), a common misdiagnosis of CSF1R‐RD. In the Hayer et al. study, MS was characterized by significantly lower NfL levels, which may indicate a faster progression of neurodegeneration in CSF1R‐RD. The first clinical symptoms may occur with a delay in relation to the pathological process, and an increase in NfL in asymptomatic or mildly symptomatic patients may indicate the initiation of the pathogenic process. This finding has profound implications for currently available treatments, such as off‐label HSCT. Available data support offering treatments only after the emergence of symptomatic disease [9]. Identifying this moment based on objective biomarkers could allow for optimization of therapy efficacy.

We also found a significant correlation between clinical severity diagnostics and NfL levels. Interestingly, in the study by Serreno et al. [31], the NfL level correlated with the degree of spasticity, fatigue, depression and MoCA; however, it is worth noting that our study was conducted on a larger cohort. It is also important to note that previous studies [31, 32] relied on scales addressing only specific aspects of the CSF1R‐RD clinical presentation. In contrast, the use of our CCSS allowed a more comprehensive assessment of the disease's overall clinical symptoms. NfL has a promising potential as a robust biomarker of CSF1R‐RD disease progression and could be used to track treatment efficacy in clinical trials. NfL is not able to differentiate CSF1R‐RD cases from other rapidly neurodegenerative diseases due to its non‐specific behavior and should be used only in patients with a genetic diagnosis of CSF1R‐RD. NfL is not typically substantially elevated in the early stages of many different glial‐cell‐related conditions and may not be sensitive to the initial phase of axonal damage [17, 18, 19, 33]. Our study confirms the utility of NfL in a larger patient cohort, further reinforcing its strong association with clinical symptom severity and structural CNS pathology. Notably, this association was supported by a novel integration of neuroimaging analysis.

For leukoencephalopathies that are primarily characterized by cellular dysfunction at the glial cell level, GFAP may better reflect the pathological process, especially in early disease stages. GFAP has already proven to be a particularly robust biomarker for X‐linked adrenoleukodystrophy, metachromatic leukodystrophy, and Alexander disease [21, 22, 34]. This biomarker has not been previously evaluated in the context of CSF1R‐RD. Our study provides the first data supporting its potential utility in clinical assessment of this disease. In our cohort, plasma GFAP was notably elevated in asymptomatic *CSF1R* pathogenic variant carriers when compared to both healthy controls and symptomatic CSF1R‐RD patients. This finding suggests that GFAP may be a robust biomarker for detecting early symptom onset, particularly compensating for NfL's known limitations in detection at this stage of disease. CSF GFAP was weakly correlated with the CCSS but did not show a correlation with MoCA, most likely due to small sample size.

Based on the strong and consistent association observed between NfL levels, clinical symptoms, and neuroimaging biomarkers in our cohort, as well as supporting evidence from previous studies [30, 31], we propose a complementary role for NfL and GFAP in the clinical management of CSF1R‐RD. While GFAP demonstrates exceptional sensitivity in distinguishing early‐stage patients from healthy controls and may help identify the optimal timing for therapeutic interventions, NfL appears to be more effective for tracking disease progression following the onset of symptoms.

This is also the first study to evaluate potential disease‐specific biomarkers that are directly implicated in the pathophysiology of CSF1R‐RD. IL‐34 is a ligand for CSF1R; therefore IL‐34 could serve as a more specific marker for CSF1R. Research on IL‐34 activation in rodent microglia and human macrophages suggests that it has distinct properties compared to colony‐stimulating factor 1 (CSF1), resulting in an anti‐inflammatory and reparative phenotype. However, chronic IL‐34 activation of microglia enhances the expression of pro‐inflammatory cytokines, contributing to neuroinflammation during the later stages of neurological disorders [23, 24, 35]. In our cohort, IL‐34 levels were elevated in all patients with pathological *CSF1R* variants compared with healthy controls, suggesting a potential role for IL‐34 in the preclinical phase of CSF1R‐RD. In our cohort, higher IL‐34 levels were observed in patients with less extensive white matter lesions, suggesting that IL‐34 may have a protective role in the preclinical stage. However, this hypothesis cannot be confirmed based on the current study.

Another potential biomarker for CSF1R‐RD is M‐CSF, which signals through its receptor, CSF1R, and thus, like IL‐34, is involved in the pathophysiology of CSF1R‐RD. Interestingly, mice deficient in *CSF1R* expression exhibit a more pronounced phenotype than those lacking M‐CSF [25]. In our cohort, M‐CSF, like IL‐34, was elevated in both asymptomatic and symptomatic carriers of pathogenic *CSF1R* variants compared with healthy controls and did not correlate with clinical severity diagnostics.

Interestingly, despite osteopontin being recognized as a marker of microglial activation [36, 37], its levels in CSF and plasma in our cohort remained largely unchanged. This finding suggests that osteopontin is not a reliable surrogate for microglial dysfunction in this specific context. One possible explanation is that the microglial pathology in CSF1R‐RD may involve functional impairment or loss rather than classical activation, which typically drives osteopontin upregulation. These observations underscore the complexity of microglial phenotypes and highlight the need for more nuanced biomarkers that can distinguish between microglial activation, degeneration, and dysfunction.

In our study, we utilized a robust cohort—representing, to our knowledge, the largest cohort of its kind to date. Most of these individuals also underwent neuroimaging biomarker testing, further enhancing the depth of our analysis.

Another strength of our study is that none of the individuals included underwent therapeutic interventions. These individuals illustrate the natural disease course, and can serve as an untreated reference point for evaluating clinical trials in CSF1R‐RD. However, our studies also present some limitations, Due to the limited sample size available for our association analyses, it was not statistically feasible to adjust for covariates such as age at biomarker collection and sex. Furthermore, this cohort lacks follow‐up data, making it unclear how useful these biomarkers would be in longitudinal cohorts. Another limitation is the lack of evaluation of CLP, which has been identified as a strong CSF1R‐RD biomarker in previous studies [31, 32].

Ultimately, our study demonstrates that the plasma biomarkers NfL and GFAP are promising tools for detecting the onset and progression of CSF1R‐RD, providing a robust means to evaluate patients and serving as a valuable reference for future clinical trials.

## Author Contributions

**Tomasz Chmiela:** methodology, software, data curation, investigation, validation, formal analysis, writing – original draft, writing – review and editing. **Madison Reeves:** methodology, software, data curation, investigation, validation, formal analysis, writing – original draft, writing – review and editing. **Karen Jansen‐West:** investigation, writing – review and editing. **Judith Dunmore:** investigation, writing – review and editing. **Yuping Song:** investigation, writing – review and editing. **Audrey Strongosky:** investigation, writing – review and editing. **Sunil Gandhi:** conceptualization, investigation, funding acquisition, writing – review and editing. **Gilana Pikover:** investigation, funding acquisition, writing – review and editing. **Matt Blurton‐Jones:** conceptualization, investigation, funding acquisition, writing – review and editing. **Robert C. Spitale:** conceptualization, investigation, funding acquisition, writing – review and editing. **Erik H. Middlebrooks:** methodology, software, data curation, investigation, validation, formal analysis, visualization, writing – original draft, writing – review and editing. **Leonard Petrucelli:** conceptualization, validation, formal analysis, supervision, funding acquisition, project administration, resources, writing – review and editing. **Mercedes Prudencio:** conceptualization, validation, formal analysis, supervision, funding acquisition, project administration, resources, writing – original draft, writing – original draft, writing – review and editing. **Zbigniew K. Wszolek:** conceptualization, validation, formal analysis, supervision, funding acquisition, project administration, resources, writing – review and editing.

## Conflicts of Interest

S.G., G.P., M.B.‐J. and R.C.S. are employed by Savanna Biotherapeutics, Z.K.W. serves as PI or Co‐PI on Biohaven Pharmaceuticals Inc. (BHV4157‐206), ONO‐2808‐03, and Amylyx AMX0035‐009 projects/grants. He serves as Co‐PI of the Mayo Clinic APDA Center for Advanced Research and as a consultant for Savanna Bio, Eli Lilly and Company. All other authors declare no conflicts of interest.

## Supporting information


**Data S1:** acn370250‐sup‐0001‐Supinfo1.docx.

**Tool S1:** CSF1R‐RD Clinical Severity Score (CCSS) diagnostic scale.

**Figure S1:** acn370250‐sup‐0003‐FigureS1‐S4‐TableS1.docx. *CSF1R* varaints detected in the CSF1R‐RD patient cohort (related to Table 1). Schematic represenation (top) of the location of the different *CSF1R* mutations (listed at the bottom) within the tyrosine kinase domain. Note cases 1–14 are asymptomatic and 15–31 are symptomatic *CSF1R* carriers.
**Figure S2:** Neuroimaging markers in asymptomatic and symptomatic *CSF1R* carriers (related to Figure 1). Sundal—atrophy (A), Sundal—white matter (B), Normalized brain volume (C), Normalized white matter volume (D), Corpus callosum total volume (E), Cerebral white matter volume (F), Subcortical gray matter volume (H), Ventricle volume (I), and Cortex volume (J), levels compared between symptomatic and asymptomatic groups using Mann Whitney *U* Tests.
**Figure S3:** The ability of CSF NfL and GFAP to discriminate between symptomatic *CSF1R* patients, asymptomatic *CSF1R* carriers, and controls (related to Figure 2). Receiver operating curves (ROC) with area under the curve (AUC) measures (with 95% confidence interval) test for CSF NfL (A) and CSF GFAP (B).
**Figure S4:** M‐CSF, IL‐34 and osteopontin levels in CSF and plasma of CSF1R‐RD cases and controls (related to Figure 2). M‐CSF CSF (A) and plasma (B), IL‐34 CSF (C) and plasma (D), and osteopontin CSF (E) and plasma (D) levels compared between symptomatic, asymptomatic, and control groups using Dunn's tests. *p*‐values < 0.0167 are considered significant after applying a Bonferroni correction for multiple testing.
**Table S1:** Summary of studies evaluating NfL, GFAP, M‐CSF, IL‐34, osteopontin, and other biomarkers in CSF1R‐RD cases.

## Data Availability

The data that support the findings of this study are available from the corresponding author upon reasonable request.
