# Supplementary material for: Fluid and Neuroimaging Biomarkers in Microgliopathy Colony‐Stimulating Factor‐1 Receptor‐Related Disorders
Source: Ann Clin Transl Neurol. 2026 Jan 12;13(6):1236–48. doi: 10.1002/acn3.70250 (PMC13251445; doi:10.1002/acn3.70250)
Supplement: Supplementary file 1 — Data S1: acn370250‐sup‐0001‐Supinfo1.docx. [file ACN3-13-1236-s003.docx]

**Supplementary Methods**

**Neurological assessment**

Every participant underwent a structured neurological assessment. We developed a semi-quantitative scale that captures all possible symptoms of CSF1R-RD (henceforth referred to as CSF1R Clinical Severity Score (CCSS)). It assesses the patient in five domains: cognition, mood and affect, cranial nerves, motor function and sensory function. For each symptom assessed, the patient was given points according to the severity of the symptom rated as mild (1 point), moderate (2 points) or severe (3 points), then the points were counted in each category and summed into a total CCSS. It is important to note that our CCSS is sensitive enough to detect even minor cognitive and motor deviations- therefore, it is possible that a patient who is classified as asymptomatic may still have points on this scale. Symptomatic CSF1R-RD patients were defined as those exhibiting clear, progressive signs or symptoms of CSF1R-RD and having a CCSS greater than 5. Patients with a CCSS of 5 or less were considered asymptomatic. The detailed scoring system can be found in **Supplementary Tool 1**. Cognitive functions were also assessed with The Montreal Cognitive Assessment (MoCA). CCSS and MoCA were then correlated with neuroimaging results and fluid biomarkers. We analyzed the total CCSS to better reflect biomarker results with the overall clinical status of CSF1R-RD patients. All research subjects both symptomatic and asymptomatic were examined by the same neurologist (ZKW).

**Neuroimaging assessment**

Twenty-three participants with *CSF1R* pathogenic variants (13 asymptomatic and 10 symptomatic) had 3-Tesla MRI of the brain available, obtained within one week of biomarker collection. Scans were analyzed by a single neuroradiologist (EHM). Sequences included in this analysis consisted of a 3D T1-weighted MPRAGE and 3D T2 FLAIR. All scans were graded using the Sundal scale ^1^, including total, white matter lesion, and atrophy scores with the reader blinded to the clinical information. White matter lesion volume was calculated from the 3D FLAIR using a deep-learning based approach implemented in Lesion Segmentation Tool (LST-AI; https://github.com/CompImg/LST-AI) ^2^ with subsequent manual verification of segmentations.

Cortical thickness data were derived for each patient from the 3D T1-weighted MPRAGE images using the default "recon-all" pipeline implemented in FreeSurfer 7.2 (http://surfer.nmr.mgh.harvard.edu). The thickness values were entered into a general linear model (mri_glmfit) for each of the primary variables of interest, including CCSS, MoCA, plasma GFAP, and plasma NfL levels. Age and sex were also entered as covariates. Permutation-based, clusterwise-corrected inference was then performed using mri_glmfit-sim (version 7.4.1) with 10,000 permutations and a clusterwise p-value threshold (cwp) of 0.05. Significant clusters were identified and characterized with MRI_surfcluster, while mri_segstats provided summary statistics for each labeled cluster. FreeSurfer segmentations were also used to derive normalized brain volume (total brain volume normalized by total intracranial volume) and corpus callosum volume.

**Biomarker assessments**

Plasma was collected from all patients and controls involved in this study, and CSF samples were collected from 16 individuals carrying pathogenic *CSF1R* variants and from all controls. Neurofilament light chains (NfL), macrophage colony stimulation factor (M-CSF), Glial fibrillary acidic protein (GFAP), interleukin-34 (IL-34) and osteopontin were assessed in both plasma and CSF.

All analytes were measured using the Simoa® HD-1 analyzer and commercially validated Simoa® HD-1 kits, Human Neurology 2-Plex assay for GFAP and NfL (Quanterix, Billerica, MA, USA). The plasma and CSF samples were assessed using 96-well plates. Each measure was repeated twice per Simoa® platform guidelines, and the mean of both measurements was provided along with the coefficient of variation (CV).

Outliers were identified by examining individual datapoints that fell above or below the 1st or 99th quantile for each biomarker measurement and removed from subsequent analyses if clinician deemed likely the result of a technical error.

**Statistical analysis**

Descriptive statistics, including mean, standard deviation, median, and interquartile range (IQR), were calculated using the ‘stats’ package in R ^3^. Differences in fluid biomarker levels across symptomatic, asymptomatic, and healthy control groups were assessed using the Kruskal-Wallis test with accompanying post-hoc Dunn’s tests. Differences in neuroimaging levels across asymptomatic and symptomatic groups were assessed using the Mann-Whitney U test. Spearman’s rank correlations were used to examine the relationships between fluid biomarkers, neuroimaging biomarkers, CCSS, and MoCA. To further visualize the relationships captured in the rank-based Spearman’s correlations, CCSS, MoCA, Total WM Lesion Volume, Normalized Brain Volume,plasma NfL, and plasma GFAP were transformed using rank-ordered quantile normalization (ORQ) and visualized in scatterplots, with linear regression models used to fit lines and test linear associations between the ranks of biomarker and CCSS, with an interaction term for patient status *^4^*. Receiver-operator characteristic curves with area under the curve (AUC) scores were generated using the ‘pROC’ package in R to compare the discriminatory ability of biomarkers of interest for patient status comparison groups *^5^*. The level of statistical significance was set at p-value < 0.05, and all tests were two-tailed. Where applicable, tests were accompanied by Bonferroni-adjusted p-value significance thresholds to account for multiple comparisons. All visualizations were generated using the ‘ggplot2’ package in R *^6^*.

1. Sundal C, Van Gerpen JA, Nicholson AM, et al. MRI characteristics and scoring in HDLS due to CSF1R gene mutations. Neurology. 2012 Aug 7;79(6):566-74.

2. CompImg. ST-AI: Deep Learning Ensemble for Accurate MS Lesion Segmentation. 1.1.0 ed: GitHub; 2024.

3. Team RC. A language and environment for statistical computing. Vienna, Austria: Foundation for Statistical Computing; 2024.

4. Peterson RA, Cavanaugh JE. Ordered quantile normalization: a semiparametric transformation built for the cross-validation era. J Appl Stat. 2020;47(13-15):2312-27.

5. Robin X, Turck N, Hainard A, et al. pROC: an open-source package for R and S+ to analyze and compare ROC curves. BMC Bioinformatics. 2011 Mar 17;12:77.

6. Wickham H. Elegant Graphics for Data Analysis. Verlag New York: Springer; 2016.
