# Supplementary material for: Fluid and Neuroimaging Biomarkers in Microgliopathy Colony‐Stimulating Factor‐1 Receptor‐Related Disorders
Source: Ann Clin Transl Neurol. 2026 Jan 12;13(6):1236–48. doi: 10.1002/acn3.70250 (PMC13251445; doi:10.1002/acn3.70250)
Supplement: Supplementary file 3 — Figure S1: acn370250‐sup‐0003‐FigureS1‐S4‐TableS1.docx. CSF1R varaints detected in the CSF1R‐RD patient cohort (related to Table 1). Schematic represenation (top) of the location of the different CSF1R mutations (listed at the bottom) within the tyrosine kinase domain. Note cases 1–14 are asymptomatic and 15–31 are symptomatic CSF1R carriers. Figure S2: Neuroimaging markers in asymptomatic and symptomatic CSF1R carriers (related to Figure 1). Sundal—atrophy (A), Sundal—white matter (B), Normalized brain volume (C), Normalized white matter volume (D), Corpus callosum total volume (E), Cerebral white matter volume (F), Subcortical gray matter volume (H), Ventricle volume (I), and Cortex volume (J), levels compared between symptomatic and asymptomatic groups using Mann Whitney U Tests. Figure S3: The ability of CSF NfL and GFAP to discriminate between symptomatic CSF1R patients, asymptomatic CSF1R carriers, and controls (related to Figure 2). Receiver operating curves (ROC) with area under the curve (AUC) measures (with 95% confidence interval) test for CSF NfL (A) and CSF GFAP (B). Figure S4: M‐CSF, IL‐34 and osteopontin levels in CSF and plasma of CSF1R‐RD cases and controls (related to Figure 2). M‐CSF CSF (A) and plasma (B), IL‐34 CSF (C) and plasma (D), and osteopontin CSF (E) and plasma (D) levels compared between symptomatic, asymptomatic, and control groups using Dunn's tests. p‐values < 0.0167 are considered significant after applying a Bonferroni correction for multiple testing. Table S1: Summary of studies evaluating NfL, GFAP, M‐CSF, IL‐34, osteopontin, and other biomarkers in CSF1R‐RD cases. [file ACN3-13-1236-s002.docx]

**Supplementary Figures and Tables**

**Suplementary Figure 1.**


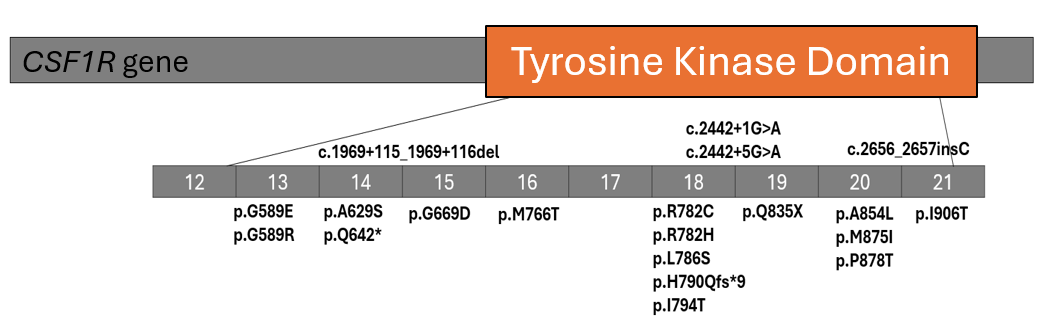


| **No.** | ***CSF1R* variant** |
| --- | --- |
| **1** | p.G589E |
| **2** | p.A629S |
| **3** | p.Q642* |
| **4** | p.G669D |
| **5** | c.1969+115_1969_116del |
| **6** | p.M766T |
| **7** | c.2442+1G>A |
| **8** | p.R782H |
| **9** | p.L786S |
| **10** | p.H790Qfs*9 |
| **11** | p.I794T |
| **12** | p.P878T |
| **13-14** | c.2656_2657insC |
| **15** | p.G589R |
| **16-17** | p.G589E |
| **18** | p.A629S |
| **19-20** | c.1969+115_1969_116del |
| **21** | p.I794T |
| **22** | c.2442+1G>A |
| **23** | c.2442+5G>A |
| **24** | p.M875I |
| **25** | p.R782C |
| **26** | p.Q835X |
| **27** | p.A854L |
| **28** | p.P878T |
| **29-31** | c.2656_2657insC |

**Supplementary Figure 1.** ***CSF1R* varaints detected in the CSF1R-RD patient cohort (related to Table 1).** Schematic represenation (top) of the location of the different *CSF1R* mutations (listed at the bottom) within the tyrosine kinase domain. Note cases 1-14 are asymptomatic and 15-31 are symptomatic *CSF1R* carriers.

**Supplementary Figure 2.**

**Supplementary Figure 2. Neuroimaging markers in asymptomatic and symptomatic *CSF1R* carriers (related to Figure 1).** Sundal – atrophy (**A**), Sundal – white matter (**B**), Normalized brain volume (**C**), Normalized white matter volume (**D**), Corpus callosum total volume (**E**), Cerebral white matter volume (**F**), Subcortical grey matter volume (**H**), Ventricle volume **(I**), and Cortex volume (**J**), levels compared between symptomatic and asymptomatic groups using Mann Whitney U Tests.

**Supplementary Figure 3.**

**Supplementary Figure 3. The ability of CSF NfL and GFAP to discriminate between symptomatic *CSF1R* patients, asymptomatic *CSF1R* carriers, and controls (related to Figure 2).** Receiver operating curves (ROC) with area under the curve (AUC) measures (with 95% confidence interval) test for CSF NfL (**A**) and CSF GFAP (**B**).

**Supplementary Figure 4.**

**Supplementary Figure 4.** **M-CSF, IL-34 and osteopontin levels in CSF and plasma of CSF1R-RD cases and controls (related to Figure 2).** M-CSF CSF (**A**) and plasma (**B**), IL-34 CSF (**C**) and plasma (**D**), and osteopontin CSF (**E**) and plasma (**D**) levels compared between symptomatic, asymptomatic, and control groups using Dunn’s tests. P-values < 0.0167 are considered significant after applying a Bonferroni correction for multiple testing.

**Supplementary Table 1. Summary of studies evaluating NfL, GFAP, M-CSF, IL-34, osteopontin, and other biomarkers in CSF1R-RD cases.**

|  | **This report** | **Hayer et al. (2018) (1)** | **Hayer et al.**  **(2022) (*2*)** | **Serreno et al.**  **(2024) (3)** |
| --- | --- | --- | --- | --- |
| No. symptomatic CSF1R-RD (n) | 17 | 10 | 14 | 11 |
| No. asymptomatic CSF1R carriers | 14 | 7 | 7 | 7 |
| No. controls | 30 | 26 | 10 | 15 |
| Studied biomarkers: | NfL  GFAP  M-CSF  IL-34  Osteopontin | NfL | CHIT | NfL  CHIT  CHI3L2 |
| Elevated in symptomatic cases (vs controls) | NfL*  GFAP***  M-CSF*  IL-34* | NfL* | CHIT* | NfL*  CHIT*  CHI3L2* |
| Elevated in asymptomatic cases (vs controls) | GFAP*  IL-34*  M-CSF* | NfL* | CHIT not elevated | CHIT  NfL |
| Correlation with clinical symptoms | NfL* (with CCSS and MoCA)  GFAP*** (with CCSS and MoCA,) | Not done | CHIT  No correlation with MoCA and Barthel Index  Negative correlation with disease duration** | CHIT and  CHI3L2 correlation with: SPRS, PHQ-8, GAD-7, FSS, MoCA, PSS, MCS, SF-12  NfL correlation with SPRS, FSS, PHQ-8, PCS, MCS |
| *Both in plasma and CSF  **Only CSF  ***Only plasma | | | | |

Summary of studies on biomarkers in CSF1R-RD. NfL- neurofilaments light chains, glial fibrillary acidic protein (GFAP), macrophage colony stimulating factor (M-CSF), interleukin-34 (IL-34), CHIT – Chitotriosidase, CHI3L2 - chitinase 3-like 2. CCSS – Colony stimulation factor-1 Receptor Related Disorder Clinical Severity Scale. *Statistically significant only in plasma. SPRS - Spastic Paraplegia Rating Scale, PHQ-8 - Patient Health Questionnaire-8, GAD-7 - Generalized Anxiety Disorder 7-item, FSS- Fatigue Severity Scale, PSS - Perceived Stress Scale, MCS - Malnutrition Care Score, SF-12 - 12-Item Short Form Survey

References:

1. S. N. Hayer, I. Krey, C. Barro, F. Rössler, P. Körtvelyessy, J. R. Lemke, J. Kuhle, L. Schöls, NfL is a biomarker for adult-onset leukoencephalopathy with axonal spheroids and pigmented glia. *Neurology* **91**, 755-757 (2018).

2. S. N. Hayer, V. Santhanakumaran, J. Böhringer, L. Schöls, Chitotriosidase is a biomarker for adult-onset leukoencephalopathy with axonal spheroids and pigmented glia. *Ann Clin Transl Neurol* **9**, 1807-1812 (2022).

3. P. L. Serrano, T. P. V. Rodrigues, L. D. Pinto, I. C. Pereira, I. B. Farias, R. B. R. Cavalheiro, P. M. Mendes, K. O. Peixoto, J. P. Barile, D. D. Seneor, E. G. Correa Silva, A. S. B. Oliveira, W. Pinto, P. Sgobbi, Assessing Chitinases and Neurofilament Light Chain as Biomarkers for Adult-Onset Leukodystrophies. *Curr Issues Mol Biol* **46**, 4309-4323 (2024).
